# Editorial: Alternatives to Antimicrobial Growth Promoters and Their Impact in Gut Microbiota, Health and Disease

**DOI:** 10.3389/fvets.2017.00196

**Published:** 2017-11-10

**Authors:** Guillermo Tellez, Juan D. Latorre

**Affiliations:** ^1^Department of Poultry Science, University of Arkansas Fayetteville, Fayetteville, AR, United States

**Keywords:** probiotics, prebiotics, phytoadditives, vector vaccine, intestinal health, animal production

**Editorial on the Research Topic**

**Alternatives to Antimicrobial Growth Promoters and Their Impact in Gut Microbiota, Health and Disease**

It has been estimated that foodborne infections in the USA cause over 76 million illnesses responsible for 5,000 fatalities each year (1). In addition, the annual economic loss attributed to the four most common enteropathogens (*Salmonella* spp., *Campylobacter* spp., *E. coli*, and *Shigella* spp.) has been estimated to reach $7 billion dollars (2). Hence, elimination of these pathogens from animal products has become a priority due to the increased numbers of human foodborne cases and governmental regulations (3). As a result, several methods to control foodborne pathogens have been implemented, including the use of antibiotics. Nevertheless, history has confirmed that the widespread use of even new antibiotics is ultimately followed, by the appearance of resistance to those drugs, creating issues at a global scale. In recent years, substantial scientific evidence has shown that the use of certain antibiotics increases enteric colonization of antibiotic-resistant strains of enteric pathogens not only in humans but also in domestic animals (4, 5). Some of these pathogens have been shown to be extremely resistant to all antibiotics commonly used, or are capable of rapidly develop resistance when exposed to antibiotic prophylaxis or treatment. As a result, an increase in the rate and severity of these infections in food-producing animals as well as in humans has been reported in many countries around the world (6–9). Antibiotics are ineffective in the treatment of multidrug resistant bacteria. Equally frighteningly, is the fact that indiscriminate use of antibiotics can actually induce disruption of the intestinal microbiome (10, 11), reducing the production of short chain fatty acids (12) and increasing luminal pH in the distal gastrointestinal tract (13). Therefore, we must reconsider the negative consequences that disruption of the microbiome has in the biology of metazoans (dysbacteriosis). A common inclination is to classify all bacteria as “harmful” entities. Nothing could be further from the truth. The number of valuable bacterial species far exceeds the number of pathogenic species and are, in reality, essential for life. After millions of years of evolution, prokaryotes established diverse interactions with eukaryotes (14) and then life on earth change. These cooperative interactions between kingdoms (mutualism) have a fundamental role in the generation and conservation of life (15, 16). One example is the gut microbiome, estimated to contain 500–1,000 different bacterial species and clearly outnumbering the total number of genes and cells of the host by an estimated of 10-fold (17). Collectively, the intestinal microbiome represents a “forgotten organ,” responsible for orchestrating major physiological tasks. Contrast with control animals, gnotobiotic animals have numerous host functions affected by the lack of intestinal microbiome, therefore affecting their immune, endocrine, nervous, and digestive systems (18–22). In simple words, both animal and plant life depend on the mutualism relationships with their related cousins, prokaryotes. And yet, the fragile composition of the microbiome is influenced by many factors such as mode of delivery, age, dietary nutrient composition, infections, inflammation, stress, and of course, medication (23, 24). It is, therefore, not surprising to see that as a result of the indiscriminate use and abuse of antibiotics, the incidence of some foodborne pathogens such as *Salmonella* and *Campylobacter* are increasing worldwide, with reports of antibiotic resistance in clinical isolates of these and other enteric pathogens (25–27). Consequently, the World Health Organization (WHO) published a list of antibiotics that should be reserved for human use only (28). Interestingly, soon after the publication of the WHO report, and with growing consumer and scientific pressures, the European Union went one step further, creating new legislations banning the use of all antibiotics as growth promoters as of January 2006 (29–31). However, in some countries, the indiscriminate use and misuse of antibiotics are still a sad reality, particularly where there is no legislation regulating the use of antibiotic in animal agriculture. Particularly in those countries, is remarkable to confirm the alarming incidence of certain enteric pathogens associated with the indiscriminate use of some antibiotics by food-producing companies (10, 32–34). Antibiotics should be limited to infections of specific bacteria with known antibiotic sensitivity.

Over a century ago, Metchnikoff (35) proposed the revolutionary idea to consume viable bacteria to promote health by modulating the intestinal microflora. The idea is more applicable now than ever since bacterial antimicrobial resistance has become a serious worldwide problem both in medical and agricultural fields. It looks like finally, we humans have learned that this is a lost war against bacterial pathogens, especially, if we keep abusing of antibiotics. Bacteria are equipped with the biological mechanisms to evolve and find mechanisms of resistance against any chemical. Hence, antibiotic alternatives such as probiotics, prebiotics, phytochemicals, enzymes, organic acids, and vaccines to improve disease resistance in highly intense/stress food animal production systems have become a priority for many scientists around the world (36, 37). Evidently, there is no such thing as a silver bullet. Rather, the combination of several of these nutraceuticals, accompanied with good husbandry and management practices, oriented to improve biosecurity programs are becoming the new strategies incorporated in many companies. In this research topic, we present 10 original research articles and 1 general commentary article included in 5 different chapters, evaluating multiple alternatives to antibiotic growth promoters to be used in animal production.

## Author Contributions

All authors listed have made a substantial, direct, and intellectual contribution to the work and approved it for publication.

## Conflict of Interest Statement

The authors declare that the research was conducted in the absence of any commercial or financial relationships that could be construed as a potential conflict of interest.
